# 1788. COVID-19 and Antibiotic Prescriptions in the United States: A County-level Analysis

**DOI:** 10.1093/ofid/ofac492.1418

**Published:** 2022-12-15

**Authors:** Alisa Hamilton, Suprena Poleon, Jerald Cherian, Sara E Cosgrove, Ramanan Laxminarayan, Eili Y Klein

**Affiliations:** Center for Disease Dynamics, Economics & Policy, Washington, District of Columbia; Center for Disease Dynamics, Economics & Policy, Washington, District of Columbia; Johns Hopkins University School of Medicine, Baltimore, Maryland; Johns Hopkins University Department of Medicine, Baltimore, Maryland; Center for Disease Dynamics, Economics & Policy, Washington, District of Columbia; Center for Disease Dynamics, Economics & Policy, Washington, District of Columbia

## Abstract

**Background:**

Declines in outpatient antibiotic prescribing were reported during the beginning of the COVID-19 pandemic in the United States; however, the overall impact of COVID-19 cases on antibiotic prescribing remains unclear.

**Methods:**

We conducted an observational, ecological study to assess the impact of COVID-19 cases and pandemic-related, non-pharmaceutical interventions (NPIs) (e.g., school closures and facemasks) on antibiotic prescribing from February to December 2020 in the US. A random effects panel regression of county-level monthly reported COVID-19 case data and corresponding systemic antibiotic prescription data from IQVIA was used. The model controlled for county demographics, NPIs, and prior years’ prescribing.

**Results:**

Total antibiotic prescriptions fell 26.1% between March and December 2020 compared to this period from 2017 to 2019. Prescribing rates dropped most among children (Figure 1). A 1% increase in county-level monthly COVID-19 cases was associated with a 0.9% increase (95% CI 0.7%, 1.1%; p< 0.01) in monthly prescriptions dispensed to adults and a 1.2% decrease (95% CI -1.7%, -0.8%; p< 0.01) in prescriptions dispensed to children (Table 1). Counties with schools open for in-person instruction were associated with a 4.4% increase (95% CI 2.3%, 6.4%; p< 0.01) in prescriptions among children compared to counties that closed schools. Internal movement restrictions and requiring facemasks were also associated with lower prescribing among children.

**Figure 1:**
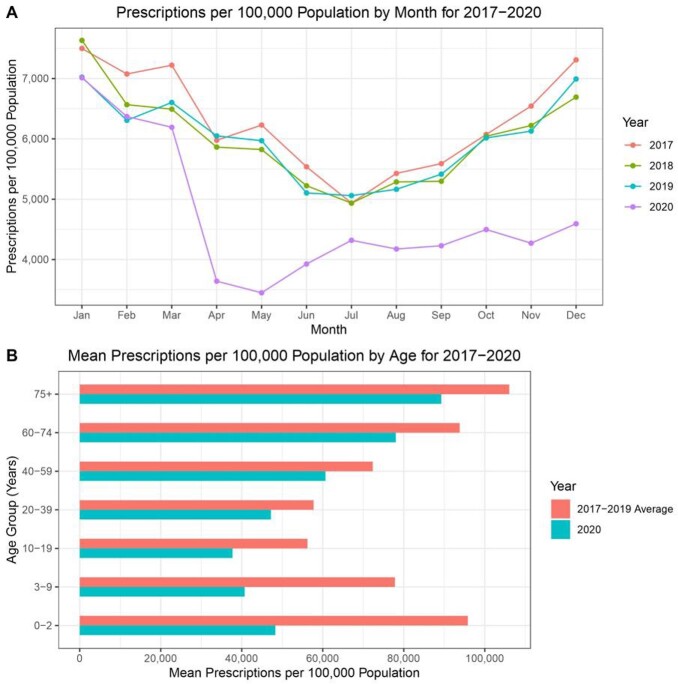
A) Total prescriptions per 100,000 population by month (2017-2020). B) Mean prescriptions per 100,000 population for seven age groups (2017-2019 vs 2020).

**Table 1:**
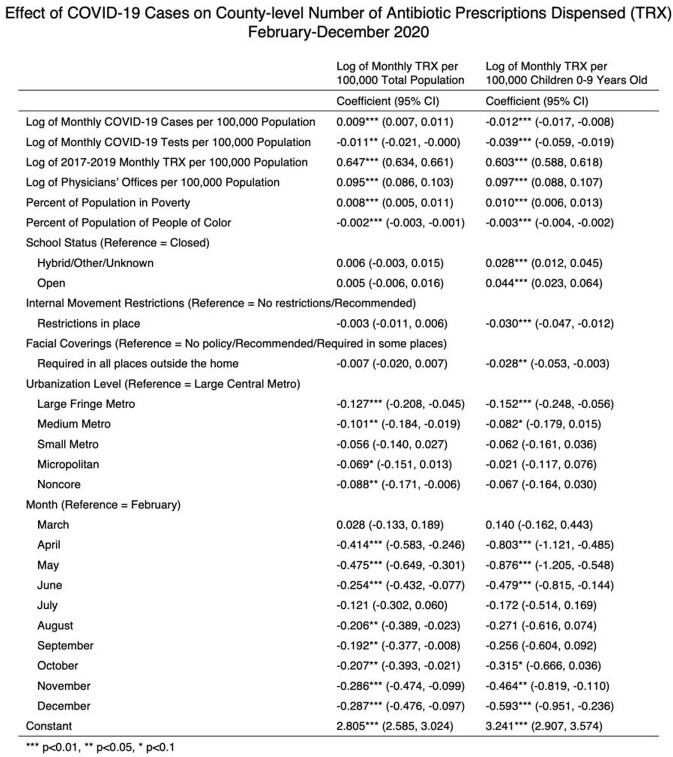

**Conclusion:**

Though the number of antibiotic prescriptions in 2020 was lower than previous years, the positive association of COVID-19 cases with prescribing for adults and the negative association for children indicates increases in prescribing occurred primarily among adults. The rarity of bacterial co-infection in COVID-19 patients suggests a large fraction of these prescriptions may have been inappropriate. Facemasks and school closures were correlated with reductions in prescribing among children, likely due to the prevention of other upper respiratory infections (e.g., the cold and influenza). Despite reductions, the strongest predictors of prescribing were prior years’ prescribing trends, suggesting the possibility that behavioral norms are an important driver of prescribing practices.

**Disclosures:**

**Sara E. Cosgrove, MD**, Basilea: Member of Infection Adjudication Committee **Ramanan Laxminarayan, PhD**, HealthCubed: Board Member|HealthCubed: Ownership Interest.

